# Guild Dynamics and Pathogen Interactions in *Hyalomma* Ticks From Algerian Cattle

**DOI:** 10.1155/tbed/5384559

**Published:** 2024-12-07

**Authors:** Salma Kaoutar Abdelali, Lynda Aissaoui, Apolline Maitre, Elianne Piloto-Sardiñas, Constance Julie, Angélique Foucault-Simonin, Sara Moutailler, Clemence Galon, Lourdes Mateos-Hernández, Dasiel Obregon, Zbigniew Zając, Alejandro Cabezas-Cruz

**Affiliations:** ^1^Department of Animal Biology and Physiology, University of Ferhat Abbas, Setif, Algeria; ^2^ANSES, INRAE, UMR BIPAR, Ecole Nationale Vétérinaire d'Alfort, Laboratoire de Santé Animale, Maisons-Alfort, France; ^3^INRAE, UR 0045 Laboratoire de Recherches Sur Le Développement de L'Elevage (SELMET-LRDE), Corte, France; ^4^Laboratoire de Virologie, Université de Corse, Corte EA 7310, France; ^5^Direction of Animal Health, National Center for Animal and Plant Health, Carretera de Tapaste y Autopista Nacional, Apartado Postal 10, San José de las Lajas 32700, Mayabeque, Cuba; ^6^School of Environmental Sciences, University of Guelph, Guelph ON, N1G 2W1, Canada; ^7^Department of Biology and Parasitology, Medical University of Lublin, Radziwiłłowska 11st, Lublin 20–080, Poland

**Keywords:** cattle, *Hyalomma*, network analysis, tick-borne pathogen interactions, ticks

## Abstract

Ticks are pivotal in transmitting a variety of pathogens that affect both humans and animals. These pathogens often occur in guilds, groups of species that exploit similar resources in similar ways. Although the composition of tick-borne pathogen (TBP) guilds is well-documented, the interactions among pathogens within these guilds remain poorly understood. We hypothesized that abiotic and biotic factors significantly influence the patterns of occurrence and interactions among pathogens within these guilds. To investigate this, we analyzed microfluidic-based high-throughput data on microorganisms from 166 *Hyalomma excavatum* ticks (94 male and 72 female) collected across different seasons from cattle in the central Algerian steppe using network analysis to uncover complex pathogen–pathogen interaction patterns. We found that female ticks had a higher infection rate (63.9%) with common pathogens such as *Rickettsia slovaca* (26.4%), unclassified Apicomplexa (22.2%), and *Borrelia afzelii* (19.4%). Male ticks showed a 56.4% infection rate, with *Rickettsia* (31.1%) and *R. slovaca* (16%) being the most prevalent. Notable pathogen–pathogen interactions within guilds were identified, with positive associations such as between *R. slovaca* and *Rickettsia conorii* in males, and *B. afzelii* and *Borrelia spielmanii* in females, indicating cooperative interactions. Conversely, negative associations, such as between *Anaplasma phagocytophilum* and *Francisella tularensis*, suggested competitive exclusion. The observed variation in interaction patterns under different conditions indicates that ecological determinants, both biotic and abiotic, influence pathogen association dynamics within guilds. These findings have significant implications for understanding disease transmission and developing control strategies.

## 1. Introduction

The rise in emerging zoonotic diseases, particularly tick-borne diseases, not only threatens public health but also has significant economic implications for the agricultural sector [1, 2]. As nearly 80% of the world's livestock is affected by ticks and the pathogens they carry, these diseases contribute to increased costs and production losses, especially in regions heavily reliant on cattle farming [3–7].

In Algeria, where cattle farming is a key industry, the prevalence of ticks, particularly those from the *Hyalomma* genus, including species like *Hyalomma anatolicum*, *Hyalomma excavatum*, and *Hyalomma marginatum*, poses a considerable challenge [8–11]. The documented presence of 24 tick species [8, 12, 13] underscores the ongoing risk to both animal and human health, reinforcing the need for integrated disease management effort. Previous studies have primarily focused on identifying these species, but fewer have explored the interactions among pathogens they transmit, which is essential for predicting outbreaks and managing disease risks.

Pathogen–pathogen interactions during coinfections can have ecological and epidemiological consequences [14], including increased virulence [15], gene transfer [16], and altered immune responses in hosts that exacerbate disease progression [17, 18]. Building on previous work [19, 20], this study uses advanced methods to explore these interactions in more detail, expanding on traditional pathogen identification by utilizing high-throughput microfluidic PCR and network analysis.

High-throughput PCR allows for simultaneous detection of multiple pathogens, offering deeper insights into pathogen diversity within individual ticks or populations than older methods like serology or standard PCR [21, 22]. Meanwhile, network analysis enables the mapping of pathogen co-occurrence and interaction patterns, which are crucial for understanding the ecological networks that shape disease transmission [23]. These tools have been successfully employed in recent studies to map tick-borne pathogen (TBP) communities across various ecosystems, revealing complex interspecies interactions [24, 25].

The ecological relevance of the ticks and pathogens is significant [21, 26]. *Hyalomma* ticks are known vectors for multiple zoonotic pathogens, including *Rickettsia* spp. and *Theileria* spp., which impact both human and animal health [5, 7]. Their feeding behavior, extended in female ticks, increases the efficiency of pathogen transmission [22]. Additionally, the microbiota of ticks influences pathogen survival and interaction, adding another layer of complexity to TBP dynamics [27]. Abiotic factors such as temperature and humidity also play a crucial role; higher temperatures have been shown to accelerate pathogen replication [28], while seasonal variations affect both tick activity and pathogen prevalence [2, 29].

This study aims to deepen the understanding of pathogen dynamics in ticks by exploring TBP guilds (TBPGs). In ecological terms, a guild refers to species that utilize the same types of resources in similar ways [30], and in TBPs, these guilds often involve pathogens sharing a host [31], such as *Hyalomma* ticks. These interactions, whether competitive or cooperative, shape community structure and influence disease transmission dynamics [32, 33]. For example, pathogens may compete for limited resources, such as access to the tick's immune system, resulting in competitive exclusion [34], or they may cooperate and enhancing each other's survival and transmission through coinfection [35].

We hypothesize that TBP interactions are influenced by tick sex, with male and female *Hyalomma* ticks showing different pathogen assemblages and interaction patterns. Additionally, seasonal changes are expected to affect TBP composition and interactions. Conducted in the Djelfa region of Algeria, this research utilizes advanced molecular techniques, including high-throughput PCR and network analysis, to provide the first comprehensive examination of TBP communities in *Hyalomma* ticks in the country. By investigating the combined effects of biotic (tick sex) and abiotic factors (seasonal changes), this study not only identifies the presence of multiple pathogens, but also reveals how their interactions within shared ecological niches (guilds) influence disease dynamics. These findings offer critical insights into the ecological and epidemiological drivers of disease transmission and the evolutionary strategies pathogens employ within the tick–host system, with significant implications for controlling tick-borne diseases in Algeria and similar regions.

## 2. Materials and Methods

### 2.1. Tick Collection

A total of 166 ticks (94 males and 72 females) were collected throughout the year 2021–2022 from 60 local breed cattle across different seasons in the province of Djelfa, Algeria (winter: 14 females and 24 males; spring: 15 females and 23 males; summer: 25 females and 23 males; autumn: 18 females and 24 males). Djelfa, positioned at 34°40′00"N and 3°15′00"E and known as the steppe capital of Algeria, provided a unique setting for this study due to its distinct environmental conditions. The semiarid climate, marked by hot summers, cold winters, sparse vegetation, and varying altitude, influences the behavior and survival of tick populations, making it an ideal location for observing how these factors affect tick–host interactions. Each cattle underwent meticulous manual inspections to ensure all ticks adhering to the skin were thoroughly removed and preserved in 70% ethanol for further analysis.

The ticks were accurately identified using a binocular magnifier (Optika, Ponteranica, Italy), with identification aided by the detailed keys from Walker et al. [36], and confirmed through sequencing of the 16S rRNA gene by molecular PCR tools. This comprehensive approach allowed for detailed study of the ticks in an environment where their natural behaviors are notably influenced by the climatic and ecological conditions of the region and confirmed through sequencing of the 16S rRNA gene by molecular PCR tools.

### 2.2. Nucleic Acid Extraction

Before extracting nucleic acids, each tick was meticulously washed with sterile milli-Q water to ensure cleanliness. DNA extraction followed, using the NucleoSpin tissue kit for Genomic DNA from tissue (Macherey-Nagel, Düren, Germany). The manufacturer's “Standard protocol for human or animal tissue and cultured cells” was employed with minor modifications tailored to our specific requirements. After disinfecting the ticks, they were carefully sectioned into quarters on a sterile petri dish using a sterile scalpel blade and then, transferred to the extraction tube that contained the provided lysis buffer. The lysis process was completed in these prefilled tubes, followed by centrifugation to separate the DNA-containing supernatant. This supernatant was then used for DNA quantification, performed with a NanoDrop spectrophotometer (Thermo Fisher Scientific, USA) at an absorbance ratio of A260/A280 to ensure purity. Finally, the extracted DNA was stored at −20°C for subsequent analyses, maintaining its integrity for future genetic examinations.

### 2.3. DNA Preamplifcation for Microfluidic Real-Time PCR

To enhance the detection of the pathogen's genetic material relative to the host's, the DNA was preamplified using the Standard BioTools preamplification kit (Standard BioTools, CA, USA). Following the manufacturer's guidelines, the process began by preparing a 0.2x pool, and then conducting PCR preamplification. Primers were combined in equal volumes to create a pooled primer mix with a final concentration of 200 nM. The preamplification reaction was performed in a 5 μl volume, comprising 1 μl of PreAmp Master Mix, 1.25 μl of the pooled primer mix, 1.5 μl of distilled water, and 1.25 μl of DNA. The thermocycling program initiated with an initial cycle at 95°C for 2 min, followed by 14 cycles of 95°C for 15 s and 60°C for 4 min. After completion, the amplification products were diluted to a 1/10th concentration and stored at −20°C to minimize contamination risks, ensuring the integrity of the samples for subsequent analysis.

### 2.4. Microfluidic Real-Time PCR Assay

Michelet et al. [37] extensively detailed the techniques utilized in their study, which focused on detecting tick-borne microorganisms. The primary method employed 48.48 Dynamic Array IFC chips (Standard BioTools, CA, USA) used within the BioMark real-time PCR system. These chips allow for the separation of 48 PCR assays and 48 samples into individual wells where real-time PCR reactions occur in separate chambers thanks to an on-chip microfluidics assembly. Each chip also includes a negative water control (Milli-Q water) to ascertain the absence of contaminants, and DNA from the *Escherichia coli* strain EDL933 (Milli-Q water and DNA diluted to 1/10) serves as an internal inhibition control in the assay plate to validate the absence of PCR inhibitors, using specific primers and a probe targeting the *E. coli* gene.

Once loaded, the BioMark real-time PCR system was programmed with parameters as reported in earlier studies [38]. Throughout this process, stringent sterility measures are maintained to ensure accurate results. Postrun analysis was conducted using the “Fluidigm Real-Time PCR Analysis” software, and results were annotated in Excel. The genes targeted and the primer sequences employed for amplification are detailed in Supporting Information 1: Table S1. This investigation cataloged a comprehensive range of tick-borne microorganisms, including 27 bacterial species such as *Borrelia burgdorferi*, *B. garinii*, *B. afzelii*, *B. valaisiana*, *B. lusitaniae*, *B. spielmanii*, *B. bissettii*, *B. miyamotoi*, *Anaplasma marginale*, *A. platys*, *A. phagocytophilum*, *A. bovis*, *A. centrale*, *A. ovis*, *Ehrlichia canis*, *N. mikurensis*, *R. conorii*, *R. slovaca*, *R. massiliae*, *R. helvetica*, *R. aeschlimannii*, *R. felis*, *Bartonella henselae*, *Francisella tularensis*, *Francisella*-like endosymbionts (FLEs), *Coxiella*-like endosymbionts (CLEs), and *Coxiella burnetii*. Additionally, seven parasite species were identified, including *Babesia microti*, *B. canis*, *B. ovis*, *B. divergens*, *B. bovis*, *B. caballi*, and *Babesia* sp. EU1. The bacterial genera included were *Bartonella*, *Borrelia*, *Anaplasma*, *Ehrlichia*, *Rickettsia*, and *Mycoplasma*, and parasite taxa encompassed Apicomplexa, *Theileria*, and *Hepatozoon*, providing a thorough overview of the pathogens present in tick populations.

### 2.5. Confirmation of Pathogen Presence Using Conventional PCR

TBPs were detected through conventional and nested PCR assays, with the cycling conditions and primers detailed in Supporting Information 2: Table S2. Additional PCR assays, utilizing species-specific primers, further confirmed the presence of certain target TBPs identified in the initial analysis. This crucial confirmation step strengthens the accuracy and reliability of the findings by providing an additional layer of validation [23].

### 2.6. DNA Sequencing Analysis

The PCR products were sequenced by Eurofins Genomics (Ebersberg, Germany), and the sequences were assembled using BioEdit software from Ibis Biosciences in Carlsbad, CA, USA. Our findings were then compared against publicly available sequences in GenBank using the online BLAST tool provided by the National Center for Biotechnology Information (NCBI, Bethesda, MD, USA), available at http://www.ncbi.nlm.nih.gov/blast.

### 2.7. Phylogenetic Analysis

A phylogenetic analysis of TBPs associated with *Hyalomma* species was performed, grouping them into 10 guilds based on collection seasons and sexs (M: males, MW: males collected in winter, MSP: males collected in spring, MSU: males collected in summer, MA: males collected in autumn, F: females, FW: females collected in winter, FSP: females collected in spring, FSU: females collected in summer, and FA: females collected in autumn). The details about pathogens identified in these TBPGs are provided in Supporting Information 3: Table S3. For this purpose, reference sequences of the 16S rRNA (bacterial pathogens) and 18S rRNA (eukaryotes) genes fragments were searched in the National Library of Medicine database; NCBI (accessed 13 June 2024). Then all sequences of particular species showing similarity to the reference ones were downloaded from the Blast database. Finally, sequences of up to 1800 nucleotides in length, excluding redundant ones underwent initial alignment using the online MAFFT tool [39]. Next, obtained set of sequences was analyzed using the MUSCLE algorithm in MEGA 11 [40]. Phylogenetic trees were then constructed using the Tamura-Nei model with Gamma distribution (TN93+G) and the Tamura 3-parameter model (T92) for the 16S rRNA and 18S rRNA gene, respectively.

Moreover, our aim was to investigate whether there is a consistent pattern of genetic distances between TBPs within each guild and whether this pattern holds across guilds. To this end, the pairwise distance between sequences within each guild was calculated (as *p*-distance) in MEGA 11. Furthermore, the statistical significance of differences in *p*-distance between the studied groups (guilds) was calculated using the Mann–Whitney *U* test, while the significance of differences in *p*-distance within particular guilds was calculated using the Wilcoxon test. Statistical calculations were performed using GraphPad 8.0 (Prism, Massachusetts, USA).

### 2.8. Statistical Analysis

The gathered data were assembled using Microsoft Excel 2016. Prevalence rates and 95% binomial confidence intervals (CIs) for each TBP infection and coinfection were calculated based on microfluidic real-time PCR amplification results. Chi-square tests (*χ*^2^) were conducted to compare TBP prevalence between males and females, a *p* value <0.05 was considered significant, the calculations were performed using SPSS software version 22.

### 2.9. Coinfections and Network Interactions Between Microorganisms

Investigations into pathogen associations within ticks have utilized a modeling approach based on binary presence/absence data. In the dataset, ticks are represented in columns and the microorganisms tested are represented in rows, where 0 indicating the absence and 1 indicating the presence of pathogen. This analysis employed Yule's *Q* statistic, defined for 2 × 2 contingency tables as:

  $$\begin{array}{c} {\text{Yule}}^{\prime }\text{s }Q=\left(ad|+|bc\right)/\left(ad|-|bc\right). \end{array}$$

*‘a'* and *‘d'* denote the number of concordant pairs (where both microorganisms are either present or absent), while *‘b'* and *‘c'* represent the number of discordant pairs (where one pathogen is present while the other is absent). Statistical analysis was conducted using the igraph package [41] implemented in R version 4.3.3 [42] and performed using RStudio [43].

Interaction networks were constructed using results from high-throughput microfluidic analyses, allowing simultaneous detection of multiple pathogens in ticks. The presence of some of these pathogens was confirmed by nested PCR. Only edges with weights of 1 and −1 were included. The resulting association networks, visualized as R plots, were constructed and refined using Gephi [44]. In each network, node color and size were indicative of modularity class and eigenvector centrality. The network's spatial layout was optimized using Yifan Hu and Fruchterman Reingold parameters within Gephi. Positive and negative interactions were determined from the correlation coefficients of abundance data. Network complexity was evaluated by examining the number of nodes, edges, and overall interaction patterns. Nodes within the network represent microorganisms, while blue and red edges denote positive and negative associations, respectively. An R script detailing the calculation of Yule's Q and the construction of the co-occurrence network is provided as additional material (Supporting Information 4: File S1).

## 3. Results

### 3.1. Tick Morphological and Genetic Classification

**Figure 1:**
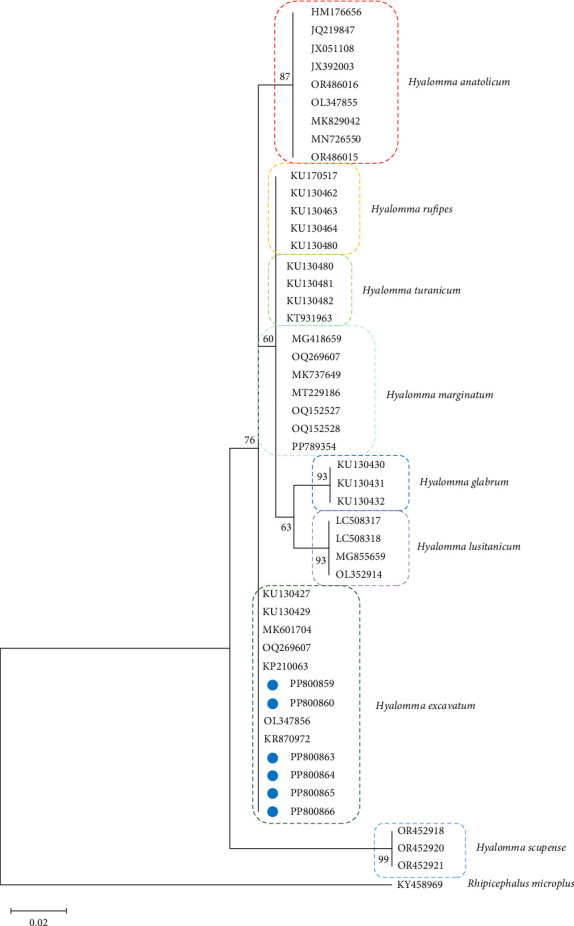
Phylogeny of the genus *Hyalomma* based on 16S rRNA gene. The evolutionary history was inferred by using the maximum likelihood method and the Tamura 3-parameter model (T92). The analysis contains sequences identified in the current study (marked with blue dot) and retrieved from GenBank database. Accession numbers of sequences are given. Bootstrap values are represented as percentage of internal branches (1000 replicates), and values lower than 50 are hidden. The tree is drawn to scale, with branch lengths measured in the number of substitutions per site. *Rhipicephalus microplus* sequence KY458969 was used to root the tree.

The ticks were morphologically identified as *H. excavatum*. To confirm this identification with higher precision, advanced PCR techniques were applied. Subsequent sequencing of the 16S rRNA gene definitively confirmed the presence of *H. excavatum*. The phylogenetic relationships of the sequences obtained further supported this identification (Figure 1). The sequences were submitted to GenBank and assigned the following accession numbers: PP800859, PP800860, PP800863, PP800864, PP800865, and PP800866. This multitiered approach of morphological examination followed by genetic verification ensured a robust classification of the tick specimens.

### 3.2. Diversity of TBPs in Ticks

The diversity of TBPs was analyzed in 166 *Hyalomma* ticks, consisting of 72 females and 94 males. Overall, 63.9% of female ticks (46/72; Table 1) and 56.4% of male ticks (53/94; Table 2) tested positive for at least one pathogen. Single infections were more common in males (45.8%, 43/94) than females (19.4%, 14/72), while coinfections were more frequent in females (44.4%, 32/72; Table 1) compared to males (10.6%, 10/94; Table 2).

Across both sexes, *Rickettsia* spp. dominated the pathogen landscape, with *R. slovaca* most prevalent in females (26.4%) and *Rickettsia* spp. highest in males (31.1%). Other notable pathogens in females included Apicomplexa (22.2%) and *Borrelia afzelii* (19.4%; Table 1), while males showed lower prevalence for Apicomplexa (5.3%) and *R. slovaca* (15.1%; Table 2).

A *χ*^2^ test (*χ*^2^ = 62.94, *p* < 0.001) confirmed significant differences in TBP diversity between sexes, suggesting distinct transmission dynamics and ecological exposures for males and females. Pathogens such as *A. phagocytophilum*, *B. afzelii*, and *B. spielmanii* were detected only in females, while *Ehrlichia* was found exclusively in males, further highlighting sex-specific pathogen associations.

### 3.3. Coinfections Between Tick-Borne Microorganisms

Coinfections were more frequent in females (44.4%, 32/72; Table 1) compared to males (10.6%, 10/94; Table 2). In females, coinfections involving two pathogens occurred in 15.3%, while coinfections of three to eight pathogens were also observed, with *R. slovaca*, *R. conorii*, and Apicomplexa being the most frequent combination (4.2%, 3/72; Table 1). In males, coinfections typically involved two pathogens (9.6%), with the most common pairing being Apicomplexa and *Rickettsia* (3.2%, 3/94; Table 2).

### 3.4. Influence of Biotic and Abiotic Ecological Determinants on Microbe–Microbe Interactions

#### 3.4.1. Tick Sex as a Biotic Ecological Determinant of Microbe–Microbe Interactions

**Figure 2:**
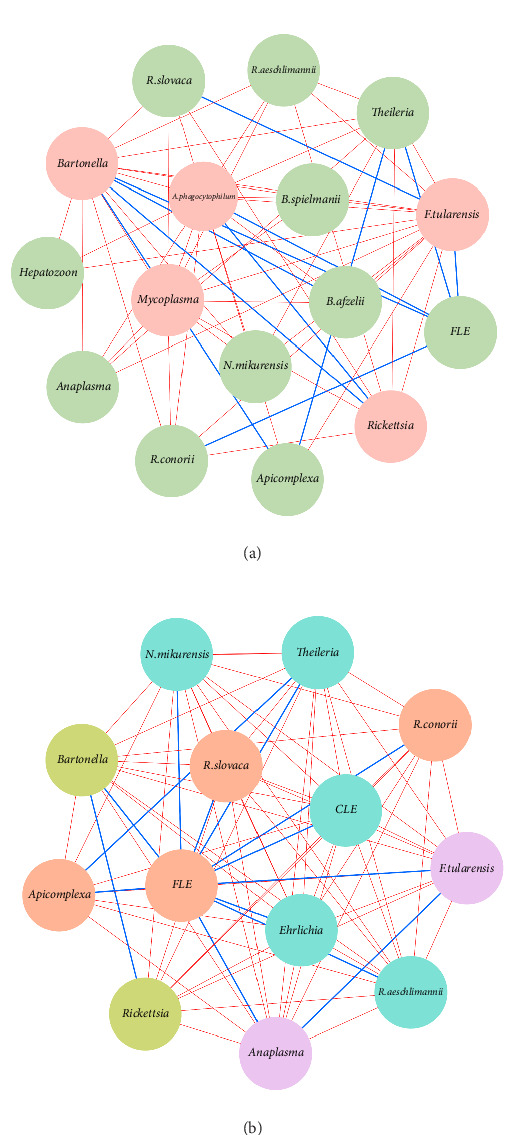
Microbial co-occurrence networks. Meaningful connections between pairs of microbial species using Yule's Q statistic: (a) female network and (b) male network. Nodes represent distinct microbial taxa, including pathogens and symbionts, while edges signify statistically significant associations with weights between 1 and −1. The colors of nodes are based on modularity class metric, and the size is proportional to the eigenvector centrality value of each taxon. Blue edges denote positive connections, while red edges represent negative ones. CLE, *Coxiella*-like endosymbionts; FLE, *Francisella*-like endosymbiont.

Network analysis of *Hyalomma* ticks revealed sex-specific pathogen interactions (Figure 2a,b). In females, negative associations between *A. phagocytophilum*, *B. afzelii*, and *F. tularensis* (Figure 2a) indicated competitive exclusion, where one pathogen's presence inhibits others. In males, strong negative interactions were found between *Anaplasma*, *Ehrlichia*, *N. mikurensis*, and *Rickettsia* species (Figure 2b), suggesting competition for resources or immune evasion strategies.

FLEs and CLEs played a central role in both sexes, showing positive associations with multiple pathogens, possibly facilitating their coexistence. Moderate positive associations, such as between *R. conorii* and *R. slovaca* in males and between *B. afzelii* and *B. spielmanii* in females, further suggest reduced competition in some coinfections.

#### 3.4.2. Seasonal Changes as an Abiotic Ecological Determinants of the Pathogen–Pathogen Interaction

**Figure 3:**
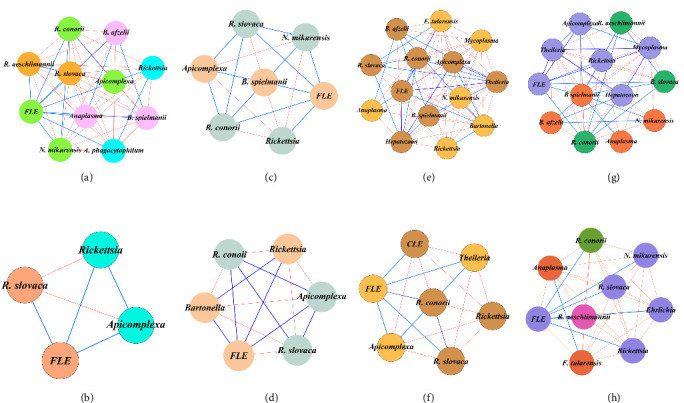
Microbial co-occurrence networks across seasons and sexes. Using co-occurrence networks, we analyzed the complex dynamics of microbe-microbe interactions in male and female Hyalomma ticks across different seasons. The figure includes separate networks for each season, presented as follows: winter networks in (a) for females and (b) for males; spring networks in (c) for females and (d) for males; summer networks in (e) for females and (f) for males; and autumn networks in (g) for females and (h) for males. The visualization showcases significant connections between pairs of microbes using Yule's Q statistic. Each node symbolizes a unique microbe, with edges indicating statistically significant associations with weights between 1 and −1. Blue edges denote positive connections, while red edges represent negative ones. The color and size of nodes reflect modularity class and eigenvector centrality, respectively. CLE, *Coxiella*-like endosymbiont; FLE, *Francisella*-like endosymbiont.

The co-occurrence networks reveal clear seasonal differences in pathogen interactions between female and male *Hyalomma* ticks (Figure 3a–h). In winter, female networks show balanced interactions between *Rickettsia* species and FLE (Figure 3a), while male networks exhibit more competitive dynamics, such as negative interactions between *R. slovaca* and Apicomplexa (Figure 3b).

In spring, female networks are more complex, dominated by positive interactions suggesting cooperation (Figure 3c), while male networks are simpler and more competitive, with taxa like *Bartonella* absent from females but present in males (Figure 3d).

In summer, females show a more diverse and complex network, with largely positive interactions and the presence of *F. tularensis* (Figure 3e), while males display stronger negative interactions, particularly between *Rickettsia* and *Theileria* (Figure 3f).

Autumn networks reflect similar patterns, with females showing more balanced interactions (Figure 3g), while males demonstrate stronger competitive pressures, particularly between species like *Anaplasma* and *R. conorii* (Figure 3h).

In pathogen–pathogen co-occurrence network of the same guild, the nodes all maintained the same value of degree centrality, suggesting the same numbers of connections for each node within the network regardless of the differences in the nature and preference of interaction (Supporting Information 5: Table S4). On the other hand, the degree centrality values of the shared nodes varied between the TBPGs networks for the same node, demonstrating that tick sex and seasonal changes influence not only the nature of interaction but also the number of associations that a taxon can establish within one condition (Supporting Information 6: Table S5).

Overall, while both female and male networks display seasonal variations in species composition and interaction patterns, males tend to exhibit more pronounced competitive interactions, particularly in summer and autumn. Females show a similarly dynamic but slightly less competitive network structure, indicating subtle differences in ecological strategies and adaptations between the sexes throughout the year.

### 3.5. Genetic Diversity and Variation in Pathogen Guilds

**Figure 4:**
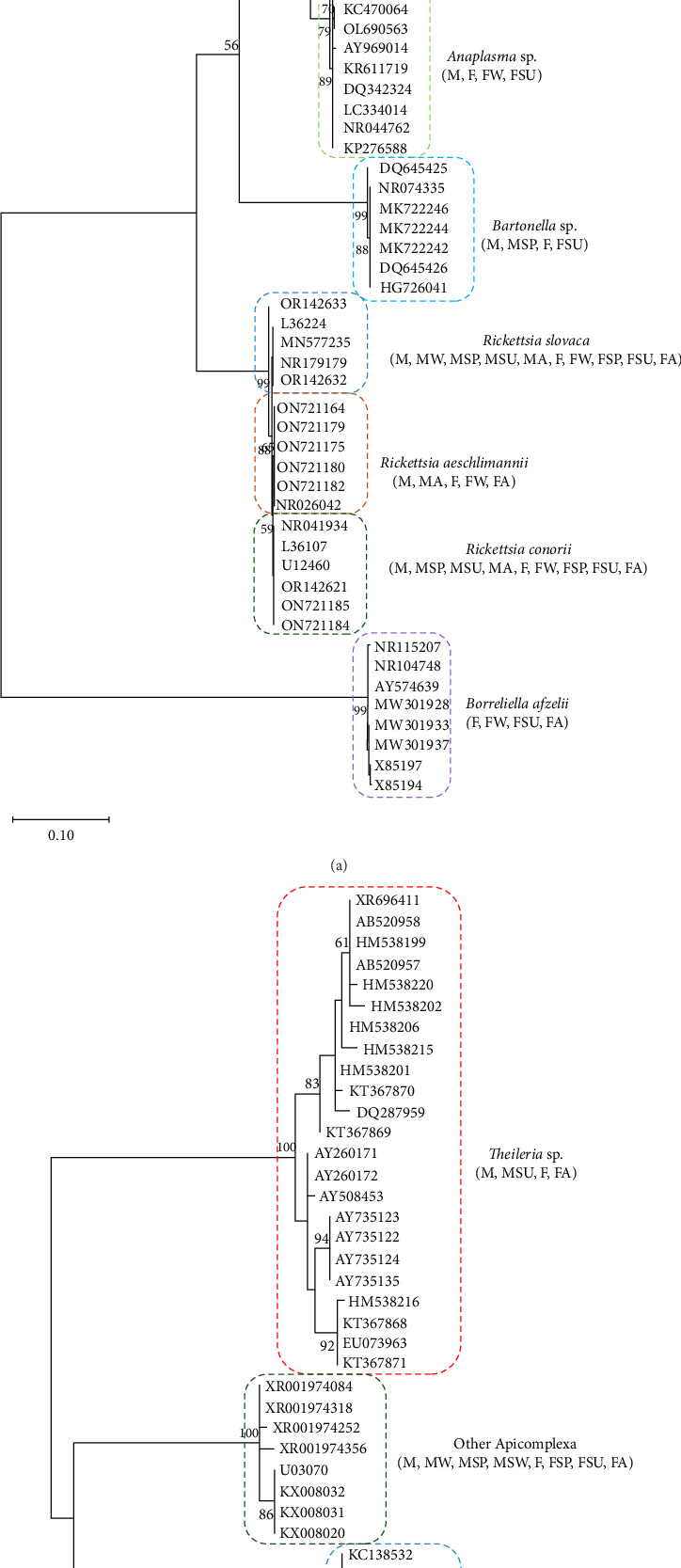
Distribution of guilds across the phylogenetic trees of tick-borne pathogens (TBPs) linked with Hyalomma excavatum. (a) Tick-borne bacteria associated with H. excavatum. The phylogram was constructed from the 16S rRNA gene, and the evolutionary history was inferred using the maximum likelihood method with the Tamura–Nei model and Gamma distribution (TN93+G). (b) Tick-borne protozoa associated with H. excavatum. The phylogram was constructed from the 18S rRNA gene, and the evolutionary history was inferred using the maximum likelihood method with the Tamura 3-parameter model (T92). For both trees, accession numbers of sequences are given. Bootstrap values are represented as percentages of internal branches (1000 replicates), with values lower than 50 hidden. The trees are drawn to scale, with branch lengths measured in the number of substitutions per site. Letters represent different guilds: F, females; FA, females collected in autumn; FSP, females collected in spring; FSU, females collected in summer; FW, females collected in winter; M, males; MA, males collected in autumn; MSP, males collected in spring; MSU, males collected in summer; MW, males collected in winter.

**Figure 5:**
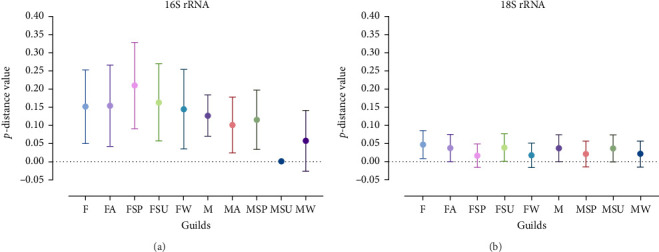
Genetic distances between sequences grouped into different guilds. (a) Genetic distances between 16S rRNA sequences grouped into different guilds. (b) Genetic distances between 18S rRNA sequences grouped into different guilds. The genetic distances were calculated as pairwise distances. The diagram shows the mean p-distance values and standard deviation ranges within each studied guild. The guilds are represented by the following abbreviations: F (females); FA (females collected in autumn); FSP (females collected in spring); FSU (females collected in summer); FW (females collected in winter); M (males); MA (males collected in autumn); MSP (males collected in spring); MSU (males collected in summer); MW (males collected in winter).

Significant genetic diversity was observed among sequences within guilds composed of bacterial pathogens (16S rRNA), surpassing that found within guilds grouping eukaryotic microorganisms (18S rRNA; Figures 4 and 5). *Rickettsia slovaca* was the only pathogen identified in all TBPGs, while *Ehrlichia* sp. was only identified in M and MA guilds (Figure 4a). The rest of the bacterial pathogens were identified in both F and M guilds and in at least one corresponding to a seasonal change guild (Figure 4a). Protozoan pathogens presented lower genetic diversity, Apicomplexa (other) was identified in a greater number and variety of guilds followed by *Hepatozoon* sp., while *Theileria* sp. was only identified in F, FSU, and FA guilds (Figure 4b).

Analysis revealed that the majority of studied guilds, with the exception of MSP 16S rRNA, displayed statistically significant variations (*p* < 0.05) in genetic distances among their constituent sequences (Figure 5, Tables 3 and 4). This trend was consistently observed across comparisons between different guilds (Figures 4 and 5 and Tables 3 and 4).

### 3.6. Confirmation of Pathogen Presence Using Conventional PCR

Utilizing conventional PCR techniques, specific genetic targets were amplified to confirm the presence of selected pathogenic species. Amplification of the 18S rRNA gene generated fragments of 1258 and 1373 bp, indicating the presence of *Babesia occultans* (accession numbers: P809771 and PP809772) in two out of nine samples tested. For *Rickettsia* species, PCR assays targeting the *gltA* and *ompB* genes produced amplicons of 282, 380, 173, and 169 bp, respectively. These results confirmed two distinct Rickettsia sequences in 2 out of 15 samples tested (PP828624 (282 bp) and PP828625 (380 bp)). Further analysis specifically identified *Rickettsia sibirica* (PP828626 (173 bp)) and *Rickettsia africae* (PP828627 (169 bp)) in 2 out of 18 samples tested. Additionally, two samples tested positive for *F. tularensis* subsp. *holarctica* via PCR in two out of two samples tested. However, sequencing of these PCR products was not attempted. The utilization of species-specific primers in these PCR assays ensured accurate identification of the target pathogens, thereby, enhancing the reliability and robustness of the study's findings.

## 4. Discussion

Traditional TBP detection methods in North Africa, like PCR and real-time PCR, are limited to identifying single pathogens [43]. Recent studies emphasize the importance of coinfections in pathogen transmission and disease severity [45–47]. This study utilizes microfluidic PCR and network analysis to examine interactions among 43 microorganisms in *Hyalomma* ticks infesting cattle in Algeria's steppe region. This innovative approach reveals the prevalence and diversity of pathogens while highlighting the complex dynamics of coinfections, providing crucial insights into pathogen community structures and their influence on disease transmission in North Africa.

One of the key findings of this study is the significant difference in pathogen prevalence and coinfection patterns between male and female ticks. These variations are likely influenced by several factors. Female ticks, which typically have longer feeding periods and consume larger blood meals compared to males, face increased exposure to pathogens [48]. Krawczyk et al. [22] suggest that this extended feeding duration, coupled with physiological differences like hormonal variations, enhances females' susceptibility to infections, such as *B. burgdorferi* and increases their likelihood of harboring multiple pathogens. Hormones like ecdysteroids and juvenile hormones, which vary between sexes, are believed to modulate immune responses and pathogen susceptibility in arthropods [48, 49]. Additionally, these physiological differences may alter the tick microbiome, potentially impacting pathogen colonization and persistence [50].

The presence of unique pathogens in female (e.g., *B. afzelii*, *B. spielmanii*, *Hepatozoon*, and *Mycoplasma*) and male (e.g., *Ehrlichia*) ticks suggests sex-specific ecological niches and behaviors that influence pathogen acquisition and transmission. These observations are consistent with findings from studies by Treuren et al. [51] and Benyedem et al. [52], which highlighted sex-specific differences in bacterial communities within ticks.

Ecological factors are crucial in the epidemiology of zoonotic diseases [53]. Climate change, marked by increased heat waves, heavy rains, and droughts, alters environmental conditions [54], affecting animal distribution and, in turn, the biology and redistribution of ticks [29]. As ticks expand, the pathogens they carry follow [2]. Tick life cycles, primarily driven by heat, rely on favorable conditions like humidity and host availability to support egg development and larval metamorphosis [55]. High temperatures can also accelerate pathogen replication, as seen with *Theileria parva*, which causes East Coast fever in cattle, while reducing transmission time in infected ticks [28]. In North Africa, *Hyalomma excavatum* is active year-round, with developmental rates peaking during warmer months [56]. This tick follows either a two- or three-host life cycle depending on host availability, adding complexity to its seasonal development [13]. Larvae and nymphs may feed on different hosts or the same one before molting into adults, creating a fluctuating landscape for pathogen transmission [36]. Seasonal peaks in tick activity often coincide with higher pathogen presence in large mammals, particularly in summer when adult ticks are most active [57].

Moreover, this study underscores the crucial role of FLEs in supporting pathogen coexistence in *Hyalomma* ticks, particularly with *Rickettsia*. FLE enhance the stability of tick microbial communities, promoting coinfections and pathogen persistence. This aligns with findings from Kumar et al. [58], who highlighted the competitive advantage of FLE over ancient endosymbionts in *Amblyomma americanum*, suggesting their ecological dominance. Azagi et al. [59] also found that imported *Hyalomma* ticks may exhibit different endosymbiont–pathogen relationships, indicating that geographical factors influence disease transmission dynamics. The evolutionary link between FLE and pathogens is further supported by Gerhart, Moses, and Raghavan [60], who showed that a FLE evolved from a mammalian pathogen, emphasizing its role in pathogen interactions. Additionally, Sesmero-García, Cabanero-Navalon, and Garcia-Bustos [61] discuss how climate change could enhance FLE's role in disease transmission, as they may help *Hyalomma* ticks adapt to changing environments.

Hussain et al. [62] propose that targeting FLE could serve as an effective tick management strategy by disrupting their symbiotic relationships, thereby, reducing tick fitness and pathogen transmission. Developing anti-microbiota vaccines to target FLE presents a promising strategy to influence tick microbiota and reduce pathogen transmission. For example, vaccination of mice against a commensal *Escherichia* in *Ixodes ricinus* altered the tick microbiota [63], leading to decreased levels of *B. afzelii* [64]. Similarly, vaccination of alpha-gal knockout mice with the same commensal decreased tick survival [65]. Additionally, microbiota-driven vaccination in soft ticks, such as *Ornithodoros moubata*, has demonstrated implications for survival, fitness, and reproductive capabilities [66]. In another study, vaccination of birds against a commensal in *Culex quinquefasciatus* effectively reduced *Plasmodium* colonization in the mosquito [67]. These results support the concept that vector microbiota manipulation by host antibodies can be utilized as a strategy to develop transmission-blocking vaccines [68].

The observed coinfections reveal important insights into disease dynamics, particularly the positive associations between pathogens like *Rickettsia conorii* and *R. slovaca* in male ticks and *B. afzelii* and *Borrelia spielmanii* in female ticks, indicating a lack of competition. Moutailler et al. [47] found a strong association between *Borrelia garinii* and *B. afzelii*, suggesting that biological interactions may promote their coinfection. Similarly, *R. conorii* and *R. slovaca* have been found to coexist without competition, as noted by Torina et al. [69]. These interactions may contribute to more complex infection patterns, influencing the epidemiology of tick-borne diseases.

Pathogens can cooperate by producing shared resources, or “common goods,” essential for their collective growth and survival. In bacterial communities, for instance, siderophores are produced to capture iron from the environment, a critical element for bacterial growth. These siderophores benefit multiple strains within the population, enhancing the overall fitness and survival of the community [70, 71]. Additionally, such cooperative behaviors are often regulated by quorum sensing, where bacteria use chemical signals to coordinate the production of these shared resources, further demonstrating the intricate cooperation among pathogens [72].

In contrast, strong negative associations between pathogens like *Anaplasma phagocytophilum* and *F. tularensis* suggest mutual exclusion. Competition among parasites within a host can lead to varied evolutionary outcomes, driven by different mechanisms [34]. Exploitation competition occurs when parasites compete for the host's limited resources by occupying overlapping ecological niches, intensifying during coinfections [73, 74]. Apparent competition, on the other hand, arises from cross-reactive immune responses, where the host's nonspecific defenses affect the abundance and success of different parasites [75, 76]. Last, interference competition involves direct suppression, where parasites actively inhibit their rivals through chemical or mechanical means [16, 77]. These competitive interactions may limit the co-occurrence of certain pathogens, impacting disease prevalence and influencing control strategies.

Interactions between pathogens in multi-infections significantly influence the evolution of virulence. Pathogens may compete for resources or cooperate to enhance survival and share resources. The observed sex-specific and seasonal variations in these interactions provide important insights into tick-borne disease dynamics. These findings highlight the need to consider both biotic and abiotic factors when developing control strategies. By combining molecular techniques with ecological and epidemiological approaches, this study enhances the understanding of TBPs and improves predictions and management strategies for their spread, leading to more effective public health interventions. While the study provides valuable insights, its findings may be constrained by the limited sample size, focus on specific tick species, and potential geographical biases. These limitations should be considered when interpreting the results and applying them to broader ecological or epidemiological contexts.

## 5. Conclusion

This study provides valuable insights into the complex interactions between TBPs within *Hyalomma excavatum* tick populations. The significant differences in pathogen prevalence and interactions between male and female ticks, along with seasonal variations, underscore the multifaceted nature of tick-borne disease ecology. These findings emphasize the need for sex-specific and seasonally tailored approaches in disease surveillance and control.

Future research should prioritize the development of targeted disease control strategies that consider seasonal and sex-based differences in tick behavior and pathogen interactions, allowing for more tailored and effective management practices. Integrating molecular diagnostics with ecological and network analyses will further advance our understanding of pathogen dynamics and support the design of innovative control strategies. One promising approach is the development of anti-microbiota vaccines, which aim to disrupt key microbial communities within ticks. By destabilizing tick microbiomes, these vaccines could reduce pathogen transmission and serve as a valuable complement to existing control measures. Emphasizing these research directions will strengthen our capacity to address the complexities of tick-borne diseases across varied ecological settings.

## Figures and Tables

**Table 1:** TBPs detected in female ticks collected from cattle using microfluidic PCR.

Vector-borne pathogen(s)	Total	Prevalence rate (%)	95% CI
Total infected ticks (≥1 pathogen)	46	63.89	51.65–74.63
*R. slovaca*	19	26.39	17.01–38.31
Apicomplexa	16	22.22	13.61–33.85
*B. afzelii*	14	19.44	11.41–30.80
Rickettsia sp.	13	18.06	10.33–29.26
*R. conorii*	12	16.67	9.27–27.70
*N. mikurensis*	11	15.28	8.23–26.12
*B. spielmanii*	10	13.89	7.22–24.52
*Anaplasma* sp.	8	11.11	5.26–21.26
*Hepatozoon* sp.	6	8.33	3.43–17.88
*R. aeschlimannii*	5	6.94	2.58–16.14
*Mycoplasma* sp.	2	2.78	0.48–10.58
*Theileria* sp.	2	2.78	0.48–10.58
*A. phagocytophilum*	1	1.39	0.07–8.54
*Bartonella* sp.	1	1.39	0.07–8.54
*F. tularensis*	1	1.39	0.07–8.54
Single infections	14	19.44	11.41–30.80
*Rickettsia* sp.	5	6.94	2.58–16.14
Apicomplexa	2	2.78	0.48–10.58
*N. mikurensis*	2	2.78	0.48–10.58
*R. slovaca*	2	2.78	0.48–10.58
*B. spielmanii*	1	1.39	0.07–8.54
*Mycoplasma* sp.	1	1.39	0.07–8.54
*Anaplasma* sp.	1	1.39	0.07–8.54
Mixed infections	32	44.44	32.90–56.59
Mixed infection with two pathogens	11	15.28	6.22–22.90
*R. slovaca* + *R. aeschlimannii*	2	2.78	0.48–10.58
*R. slovaca* + *R. conorii*	1	1.39	0.07–8.54
*R. slovaca* + Apicomplexa	1	1.39	0.07–8.55
*Rickettsia* sp. + *B. spielmanii*	1	1.39	0.07–8.54
*B. afzelii* + *B. spielmanii*	1	1.39	0.07–8.55
*A. phagocytophilum* + *Rickettsia* sp.	1	1.39	0.07–8.56
*B. afzelii* + *Rickettsia* sp.	1	1.39	0.07–8.57
*N. mikurensis* + Apicomplexa	1	1.39	0.07–8.58
*R. slovaca* + *F. tularensis*	1	1.39	0.07–8.59
*B. afzelii* + *Anaplasma* sp.	1	1.39	0.07–8.60
Mixed infection with three pathogens	9	12.50	6.22–22.90
*R. slovaca* + *R. conorii* + Apicomplexa	3	4.17	1.08–12.50
*R. slovaca* + *R. conorii* + *N. mikurensis*	1	1.39	0.07–8.54
*N. mikurensis* + Apicomplexa + *R. conorii*	1	1.39	0.07–8.55
*R. slovaca* + *R. conorii* + *Anaplasma* sp.	1	1.39	0.07–8.56
*B. afzelii* + *B. spielmanii* + *R. slovaca*	1	1.39	0.07–8.57
*N. mikurensis* + Apicomplexa + *Rickettsia* sp.	1	1.39	0.07–8.58
*R. slovaca* + *R. conorii* + *B. afzelii*	1	1.39	0.07–8.54
Mixed infection with four pathogens	6	8.33	3.43–17.88
*Rickettsia* sp. + *Bartonella* sp. + *B. afzelii* + Apicomplexa	1	1.39	0.07–8.54
*R. slovaca* + *R. conorii* + *R. aeschlimannii* + *Hepatozoon* sp.	1	1.39	0.07–8.55
*B. afzelii* + *B. spielmanii* + *Rickettsia* sp. + *Anaplasma* sp.	1	1.39	0.07–8.56
*B. afzelii* + *B. spielmanii* + *Anaplasma* sp. + Apicomplexa	1	1.39	0.07–8.57
Apicomplexa + *Mycoplasma* sp. + *Theileria* sp. + *Hepatozoon* sp.	1	1.39	0.07–8.58
*B. afzelii* + *N. mikurensis* + *Rickettsia* sp. + Hepatozoon sp.	1	1.39	0.07–8.59
Mixed infection with five pathogens	4	5.56	1.79–14.35
*B. afzelii* + Anaplasma sp. + *N. mikurensis* + Rickettsia sp. + Apicomplexa	1	1.39	0.07–8.54
*R. slovaca* + *B. spielmanii* + *R. conorii* + Apicomplexa + Hepatozoon sp.	1	1.39	0.07–8.55
*B. afzelii* + Anaplasma sp. + *N. mikurensis* + *R. aeschlimannii* + Hepatozoon sp.	1	1.39	0.07–8.56
*R. slovaca* + *R. conorii* + *B. afzelii* + *B. spielmanii* + *N. mikurensis*	1	1.39	0.07–8.57
Mixed infection with six pathogens	1	1.39	0.07–8.58
*R. slovaca* + *R. conorii* + *B. afzelii* + *B. spielmanii* + Theileria sp. + Apicomplexa	1	1.39	0.07–8.59
Mixed infection with eight pathogens	1	1.39	0.07–8.60
Hepatozoon sp. + Apicomplexa + *R. slovaca* + *R. aeschlimannii* + *B. afzelii* + *B. spielmanii* + Anaplasma sp.	1	1.39	0.07–8.61
Not detected	26	36.11	25.37–48.35

Abbreviations: CI, confidence interval; TBPs, tick-borne pathogens.

**Table 2:** TBPs detected in male ticks collected from cattle using microfluidic PCR.

Vector-borne pathogen(s)	Total	Prevalence rate (%)	95% CI
Total infected ticks (≥1 pathogen)	53	56.38	45.78–66.46
*Rickettsia* sp.	30	31.91	22.89–42.44
*R. slovaca*	15	15.96	9.5–25.27
*R. conorii*	6	6.38	2.62–13.91
Apicomplexa	5	5.32	1.97–12.55
*Anaplasma* sp.	1	1.06	0.06–6.62
*Bartonella* sp.	1	1.06	0.06–6.62
*Ehrilichia* sp.	1	1.06	0.06–6.62
*F. tularensis*	1	1.06	0.06–6.62
*N. mikurensis*	1	1.06	0.06–6.62
*R. Aeschlimannii*	1	1.06	0.06–6.62
*Theileria* sp.	1	1.06	0.06–6.62
Single infections	43	45.74	35.54–56.3
*Rickettsia* sp.	27	28.72	20.09–39.12
*R. slovaca*	11	11.70	6.27–20.38
*R. conorii*	2	2.13	0.37–8.21
*Ehrilichia* sp.	1	1.06	0.06–6.62
*N. mikurensis*	1	1.06	0.06–6.62
*R. aeschlimannii*	1	1.06	0.06–6.62
Mixed infections	10	10.64	5.5–19.12
Mixed infection with two pathogens	9	9.57	4.74–17.85
Apicomplexa *+ Rickettsia* sp.	3	3.19	0.83–9.71
*R. slovaca + R. conorii*	3	3.19	0.83–9.71
Apicomplexa *+ Theleiria*	1	1.06	0.06–6.62
*Anaplasma* sp. *+ F. tularensis*	1	1.06	0.06–6.62
*Bartonella* sp. *+ Rickettsia* sp.	1	1.06	0.06–6.62
Mixed infection with three pathogens	1	1.06	0.06–6.62
Apicomplexa *+ R. slovaca + R. conorii*	1	1.06	0,06–6.62
Not detected	41	43.62	33.54–54.22

Abbreviations: CI, confidence interval; TBPs, tick-borne pathogens.

**Table 3:** Statistical significance of genetic distances calculated as pairwise distance between particular 16 rRNA sequences grouped into guilds.

Guilds	Guilds and *p* values								
	F	FA	FSP	FSU	FW	M	MA	MSP	MSU
FA	0.862	—	—	—	—	—	—	—	—
FSP	<0.001⁣^*∗*^	<0.001⁣^*∗*^	—	—	—	—	—	—	—
FSU	0.154	0.185	<0.001⁣^*∗*^	—	—	—	—	—	—
FW	0.131	0.124	<0.001⁣^*∗*^	0.004⁣^*∗*^	—	—	—	—	—
M	0.009⁣^*∗*^	0.007⁣^*∗*^	<0.001⁣^*∗*^	<0.001⁣^*∗*^	0.525	—	—	—	—
MA	0.003⁣^*∗*^	<0.001⁣^*∗*^	<0.001⁣^*∗*^	<0.001⁣^*∗*^	0.036⁣^*∗*^	0.211	—	—	—
MSP	0.049⁣^*∗*^	0.041⁣^*∗*^	<0.001⁣^*∗*^	0.814	0.099	<0.001⁣^*∗*^	0.3811	—	—
MSU	<0.001⁣^*∗*^	<0.001⁣^*∗*^	<0.001⁣^*∗*^	<0.001⁣^*∗*^	<0.001⁣^*∗*^	<0.001⁣^*∗*^	<0.001⁣^*∗*^	<0.001⁣^*∗*^	—
MW	0.008⁣^*∗*^	0.005⁣^*∗*^	<0.001⁣^*∗*^	0.002⁣^*∗*^	0.009⁣^*∗*^	0.012⁣^*∗*^	0.001⁣^*∗*^	0.002⁣^*∗*^	0.189

*Note: p*-level of statistical significance.

Abbreviations: F, females; FA, females collected in autumn; FSP, females collected in spring; FSU, females collected in summer; FW, females collected in winter; M, males; MA, males collected in autumn; MSP, males collected in spring; MSU, males collected in summer; MW, males collected in winter.

⁣^*∗*^Statistically significant.

**Table 4:** Statistical significance of genetic distances calculated as pairwise distance between particular 18S rRNA sequences grouped into guilds.

Guilds	Guilds and *p*-Values							
	F	FA	FSP	FSU	FW	M	MSP	MSU
FA	0.006⁣^*∗*^	—	—	—	—	—	—	—
FSP	<0.001⁣^*∗*^	<0.001⁣^*∗*^	—	—	—	—	—	—
FSU	0.010⁣^*∗*^	0.753	<0.001⁣^*∗*^	—	—	—	—	—
FW	<0.001⁣^*∗*^	<0.001⁣^*∗*^	0.887	<0.001⁣^*∗*^	—	—	—	—
M	0.005⁣^*∗*^	0.904	<0.001⁣^*∗*^	0.665	<0.001⁣^*∗*^	—	—	—
MSP	<0.001⁣^*∗*^	<0.001⁣^*∗*^	0.534	<0.001⁣^*∗*^	0.627	<0.001⁣^*∗*^	—	—
MSU	0.003⁣^*∗*^	0.816	<0.001⁣^*∗*^	0.591	<0.001⁣^*∗*^	0.913	<0.001⁣^*∗*^	—
MW	<0.001⁣^*∗*^	<0.001⁣^*∗*^	0.5344	<0.001⁣^*∗*^	0.6275	<0.001⁣^*∗*^	0.999	<0.001⁣^*∗*^

*Note: p*-level of statistical significance.

Abbreviations: F, females; FA, females collected in autumn; FSP, females collected in spring; FSU, females collected in summer; FW, females collected in winter; M, males; MA, males collected in autumn; MSP, males collected in spring; MSU, males collected in summer; MW, males collected in winter.

⁣^*∗*^Statistically significant.

## Data Availability

The data that supports the findings of this study are available in the Supporting Information of this article.

## Supporting Information

Supporting Information 1 Table S1: List of primer/probe sets used in the BioMark real-time PCR system.

Supporting Information 2 Table S2: Primer pairs and PCR conditions for selected pathogens and tick species confirmation.

Supporting Information 3 Table S3: Tick-borne microorganisms identified in TBPGs associated with Hyalomma excavatum.

Supporting Information 4 File S1: R script detailing the calculation of Yule's Q and the construction of the co-occurrence network.

Supporting Information 5 Table S4: Centrality measures of pathogens in TBPGs networks.

Supporting Information 6 Table S5: Centrality measures of shared pathogens across TBPGs networks.
