# Supplementary material for: Guild Dynamics and Pathogen Interactions in Hyalomma Ticks From Algerian Cattle
Source: Transbound Emerg Dis. 2024 Dec 7;2024:5384559. doi: 10.1155/tbed/5384559 (PMC12016691; doi:10.1155/tbed/5384559)
Supplement: Supporting Information 2 — Table S2: Primer pairs and PCR conditions for selected pathogens and tick species confirmation. [file 5384559.f2.docx]

**Supplementary Table S2.** Primer pairs and PCR conditions for selected pathogens and tick species confirmation.

| Target Species/Pathogens | Primers sequences (5´- 3´) | Target gene or intergenic region | Amplicon size | PCR conditions | References |
| --- | --- | --- | --- | --- | --- |
| *Hyalomma excavatum* | TTT GAC TAT ACA AAG GTA TTG | 16S rRNA | - | 35 cycles:  10 sec 98°C; 30 sec 51.4°C; 30 sec 72°C | Roth et al. (2019) |
|  | CGG TCT GAA CTC AGA TCA AGT AGG |  |  |  |  |
| *Babesia* spp.*/Hepatozoon* spp. */Theileria* spp*.* | PCR 1 |  |  | 35 cycles: |  |
|  | GTGAAACTGCGAATGGCTCATTAC | 18S rRNA | 1500 bp | 10 sec 98°C; 30 sec 58°C; 45 sec 72°C | Masatani et al. (2017) |
|  | AAGTGATAAGGTTCACAAAACTTCCC |  |  |  |  |
|  | PCR 2 |  |  | 35 cycles: |  |
|  | GGCTCATTACAACAGTTATAGTTTATTTG |  | 1500 bp | 10 sec 98°C; 30 sec 58°C; 30 sec 72°C |  |
|  | CGGTCCGAATAATTCACCGGAT |  |  |  |  |
| *Francisella tularensis* subsp. *holarctica* | CTTGTACTTTTATTTGGCTACTGAGAAACT | A junction between ISFtu2 and a flanking 3′ region | 70- 150 Bp | 2mn 50°C  10mn 95°C  45 cycles :  10 sec 95°C; 30 sec 65°C  5mn 45°C | Kugler et al. (2006) |
|  | CTTGCTTGGTTTGTAAATATAGTGGAA |  |  |  |  |
| *Rickettsia* *sibirica/R.africae* | PCR 1 |  |  | 35 cycles: |  |
|  | GTCAGCGTTACTTCTTCGATGC | *ompB* | 475 bp | 10 sec 98°C; 30 sec 57°C; 30 sec 72°C | Choi et al. (2005) |
|  | CCGTACTCCATCTTAGCATCAG |  |  |  |  |
|  | PCR 2 |  |  | 35 cycles: |  |
|  | CCAATGGCAGGACTTAGCTACT |  | 267 bp | 10 sec 98°C; 30 sec 58°C; 30 sec 72°C |  |
|  | AGGCTGGCTGATACACGGAGTAA |  |  |  |  |
| *Rickettsia* spp. | PCR 1 |  |  | 35 cycles: |  |
|  | GGG GGC CTG CTC ACG GCG G | *gtlA* | 381 bp | 10 sec 98°C; 30 sec 56°C; 30 sec 72°C | Regnery et al. (1991) |
|  | ATT GCA AAA AGT ACA GTG AAC A |  |  |  |  |
