# Supplementary material for: Guild Dynamics and Pathogen Interactions in Hyalomma Ticks From Algerian Cattle
Source: Transbound Emerg Dis. 2024 Dec 7;2024:5384559. doi: 10.1155/tbed/5384559 (PMC12016691; doi:10.1155/tbed/5384559)
Supplement: Supporting Information 3 — Table S3: Tick-borne microorganisms identified in TBPGs associated with Hyalomma excavatum. [file 5384559.f3.docx]

**Supplementary Table S3.** Pathogens Identified in Tick-Borne Pathogen Guilds (TBPGs).

| TBPG Identifier | Tick Species | Pathogen (TBP) | Genetic Marker(s) Used | Accession Number(s) | Notes |
| --- | --- | --- | --- | --- | --- |
| M | *Hyalomma excavatum* | *Anaplasma* | 16S rRNA |  |  |
|  |  | Apicomplexa | 18S rRNA |  |  |
|  |  | *Bartonella* | *ssrA* |  |  |
|  |  | *Coxiella like endosymbiont* | *IS1111* |  |  |
|  |  | *Ehrilichia* | 16S rRNA |  |  |
|  |  | *Francisella Like endosymbiont* | *fopA* |  |  |
|  |  | *Francisella tularensis* | *tul4+* |  |  |
|  |  | *Neoehrlichia mikurensis* | *groEL* |  |  |
|  |  | *Rickettsia aeschlimannii* | *ITS* |  |  |
|  |  | *Rickettsia conorii* | *ITS* |  |  |
|  |  | *Rickettsia slovaca* | *ITS* |  |  |
|  |  | *Rickettsia* | *gltA* |  |  |
|  |  | *Theleiria* | 18S rRNA |  |  |
| MW | *Hyalomma excavatum* | Apicomplexa | 18S rRNA |  |  |
|  |  | *Francisella Like endosymbiont* | *fopA* |  |  |
|  |  | *Rickettsia slovaca* | *ITS* |  |  |
|  |  | *Rickettsia* | *gltA* |  |  |
| MSP | *Hyalomma excavatum* | Apicomplexa | *18S rRNA* |  |  |
|  |  | *Bartonella* | *ssrA* |  |  |
|  |  | *Francisella Like endosymbiont* | *fopA* |  |  |
|  |  | *Rickettsia conorii* | *ITS* |  |  |
|  |  | *Rickettsia slovaca* | *ITS* |  |  |
|  |  | *Rickettsia* | *gltA* |  |  |
| MSU | *Hyalomma excavatum* | Apicomplexa | 18S rRNA |  |  |
|  |  | *Coxiella like endosymbiont* | *IS1111* |  |  |
|  |  | *Rickettsia conorii* | *ITS* |  |  |
|  |  | *Francisella Like endosymbiont* | *fopA* |  |  |
|  |  | *Rickettsia slovaca* | *ITS* |  |  |
|  |  | *Rickettsia* | *gltA* |  |  |
|  |  | *Theleiria* | 18S rRNA |  |  |
| MA | *Hyalomma*  *Excavatum* | Apicomplexa | 18S rRNA |  |  |
|  |  | *Ehrilichia* | 16S rRNA |  |  |
|  |  | *Francisella Like endosymbiont* | *fopA* |  |  |
|  |  | *Francisella tularensis* | *tul4+* |  |  |
|  |  | *Neoehrlichia mikurensis* | *groEL* |  |  |
|  |  | *Rickettsia aeschlimannii* | *ITS* |  |  |
|  |  | *Rickettsia conorii* | *ITS* |  |  |
|  |  | *Rickettsia slovaca* | *ITS* |  |  |
|  |  | *Rickettsia* | *gltA* |  |  |
| F | *Hyalomma excavatum* | *Anaplasma phagocytophilum* | *msp2* |  |  |
|  |  | *Anaplasma* | 16S rRNA |  |  |
|  |  | Apicomplexa | 18S rRNA |  |  |
|  |  | *Bartonella* | *ssrA* |  |  |
|  |  | *Borrelia afzelii* | *Fla* |  |  |
|  |  | *Borrelia spielmanii* | *Fla* |  |  |
|  |  | *Francisella Like endosymbiont* | *fopA* |  |  |
|  |  | *Francisella tularensis* | *tul4+* |  |  |
|  |  | *Hepatozoon* | 18S rRNA |  |  |
|  |  | *Mycoplasma* | 16S rRNA |  |  |
|  |  | *Neoehrlichia mikurensis* | *groEL* |  |  |
|  |  | *Rickettsia aeschlimannii,* | *ITS* |  |  |
|  |  | *Rickettsia conorii* | *ITS* |  |  |
|  |  | *Rickettsia slovaca* | *ITS* |  |  |
|  |  | *Rickettsia* | *gltA* |  |  |
|  |  | *Theleiria* | 18S rRNA |  |  |
| FW | *Hyalomma excavatum* | *Anaplasma phagocytophilum* | *msp2* |  |  |
|  |  | *Anaplasma* | 16S rRNA |  |  |
|  |  | Apicomplexa | 18S rRNA |  |  |
|  |  | *Borrelia afzelii* | *Fla* |  |  |
|  |  | *Borrelia spielmanii* | *Fla* |  |  |
|  |  | *Francisella Like endosymbiont* | *fopA* |  |  |
|  |  | *Neoehrlichia mikurensis* | *groEL* |  |  |
|  |  | *Rickettsia aeschlimannii,* | *ITS* |  |  |
|  |  | *Rickettsia conorii* | *ITS* |  |  |
|  |  | *Rickettsia slovaca* | *ITS* |  |  |
|  |  | *Rickettsia* | *gltA* |  |  |
| FSP | *Hyalomma excavatum* | Apicomplexa | 18S rRNA |  |  |
|  |  | *Borrelia spielmanii* | *Fla* |  |  |
|  |  | *Francisella Like endosymbiont* | *fopA* |  |  |
|  |  | *Neoehrlichia mikurensis* | *groEL* |  |  |
|  |  | *Rickettsia conorii* | *ITS* |  |  |
|  |  | *Rickettsia slovaca* | *ITS* |  |  |
|  |  | *Rickettsia* | *gltA* |  |  |
| FSU | *Hyalomma excavatum* | *Anaplasma* | 16S rRNA |  |  |
|  |  | Apicomplexa | 18S rRNA |  |  |
|  |  | *Bartonella* | *ssrA* |  |  |
|  |  | *Borrelia afzelii* | *Fla* |  |  |
|  |  | *Borrelia spielmanii* | *Fla* |  |  |
|  |  | *Francisella Like endosymbiont* | *fopA* |  |  |
|  |  | *Francisella tularensis* | *tul4+* |  |  |
|  |  | *Hepatozoon* | 18S rRNA |  |  |
|  |  | *Mycoplasma* | 16S rRNA |  |  |
|  |  | *Neoehrlichia mikurensis* | *groEL* |  |  |
|  |  | *Rickettsia conorii* | *ITS* |  |  |
|  |  | *Rickettsia slovaca* | *ITS* |  |  |
|  |  | *Rickettsia* | *gltA* |  |  |
|  |  | *Theleiria* | *18S rRNA* |  |  |
| FA | *Hyalomma excavatum* | *Anaplasma* | *16S rRNA* |  |  |
|  |  | Apicomplexa | *18S rRNA* |  |  |
|  |  | *Borrelia afzelii* | *Fla* |  |  |
|  |  | *Borrelia spielmanii* | *Fla* |  |  |
|  |  | *Francisella Like endosymbiont* | *fopA* |  |  |
|  |  | *Hepatozoon* | 18S rRNA |  |  |
|  |  | *Mycoplasma* | 16S rRNA |  |  |
|  |  | *Neoehrlichia mikurensis* | *groEL* |  |  |
|  |  | *Rickettsia aeschlimannii* | *ITS* |  |  |
|  |  | *Rickettsia conorii* | *ITS* |  |  |
|  |  | *Rickettsia slovaca* | *ITS* |  |  |
|  |  | *Rickettsia* | *gltA* |  |  |
|  |  | *Theleiria* | 18S rRNA |  |  |
