# Supplementary material for: Guild Dynamics and Pathogen Interactions in Hyalomma Ticks From Algerian Cattle
Source: Transbound Emerg Dis. 2024 Dec 7;2024:5384559. doi: 10.1155/tbed/5384559 (PMC12016691; doi:10.1155/tbed/5384559)
Supplement: Supporting Information 5 — Table S4: Centrality measures of pathogens in TBPGs networks. [file 5384559.f5.docx]

**Supplementary Table S4.** Centrality measures of pathogens in Tick-Borne Pathogen Guilds (TBPGs).

| TBPG Identifier | Pathogen (TBP) | Degree Centrality | Betweenness Centrality | Eigenvector Centrality | Closeness Centrality |
| --- | --- | --- | --- | --- | --- |
| M | *Anaplasma* | 11 | 0 | 1 | 1 |
|  | Apicomplexa | 11 | 0 | 1 | 1 |
|  | *Bartonella* | 11 | 0 | 1 | 1 |
|  | *Ehrilichia* | 11 | 0 | 1 | 1 |
|  | *Francisella like endosymbiont* | 11 | 0 | 1 | 1 |
|  | *Francisella tularensis* | 11 | 0 | 1 | 1 |
|  | *Neoehrlichia mikurensis* | 11 | 0 | 1 | 1 |
|  | *Rickettsia aeschlimannii* | 11 | 0 | 1 | 1 |
|  | *Rickettsia conorii* | 11 | 0 | 1 | 1 |
|  | *Rickettsia slovaca* | 11 | 0 | 1 | 1 |
|  | *Rickettsia* | 11 | 0 | 1 | 1 |
|  | *Theleiria* | 11 | 0 | 1 | 1 |
| MW | Apicomplexa | 3 | 0 | 1 | 1 |
|  | *Francisella like endosymbiont* | 3 | 0 | 1 | 1 |
|  | *Rickettsia slovaca* | 3 | 0 | 1 | 1 |
|  | *Rickettsia* | 3 | 0 | 1 | 1 |
| MSP | Apicomplexa | 5 | 0 | 1 | 1 |
|  | *Bartonella* | 5 | 0 | 1 | 1 |
|  | *Francisella like endosymbiont* | 5 | 0 | 1 | 1 |
|  | *Rickettsia conorii* | 5 | 0 | 1 | 1 |
|  | *Rickettsia slovaca* | 5 | 0 | 1 | 1 |
|  | *Rickettsia* | 5 | 0 | 1 | 1 |
| MSU | Apicomplexa | 6 | 0 | 1 | 1 |
|  | *Coxiella like endosymbiont* | 6 | 0 | 1 | 1 |
|  | *Francisella like endosymbiont* | 6 | 0 | 1 | 1 |
|  | *Rickettsia conorii* | 6 | 0 | 1 | 1 |
|  | *Rickettsia slovaca* | 6 | 0 | 1 | 1 |
|  | *Rickettsia* | 6 | 0 | 1 | 1 |
|  | *Theleiria* | 6 | 0 | 1 | 1 |
| MA | Anaplasma | 8 | 0 | 1 | 1 |
|  | *Ehrilichia* | 8 | 0 | 1 | 1 |
|  | *Francisella like endosymbiont* | 8 | 0 | 1 | 1 |
|  | *Francisella tularensis* | 8 | 0 | 1 | 1 |
|  | *Neoehrlichia mikurensis* | 8 | 0 | 1 | 1 |
|  | *Rickettsia aeschlimannii* | 8 | 0 | 1 | 1 |
|  | *Rickettsia conorii* | 8 | 0 | 1 | 1 |
|  | *Rickettsia slovaca* | 8 | 0 | 1 | 1 |
|  | *Rickettsia* | 8 | 0 | 1 | 1 |
| F | *Anaplasma phagocytophilum* | 15 | 0 | 1 | 1 |
|  | *Anaplasma* | 15 | 0 | 1 | 1 |
|  | Apicomplexa | 15 | 0 | 1 | 1 |
|  | *Bartonella* | 15 | 0 | 1 | 1 |
|  | *Borrelia afzelii* | 15 | 0 | 1 | 1 |
|  | *Borrelia spielmanii* | 15 | 0 | 1 | 1 |
|  | *Francisella like endosymbiont* | 15 | 0 | 1 | 1 |
|  | *Francisella tularensis* | 15 | 0 | 1 | 1 |
|  | *Hepatozoon* | 15 | 0 | 1 | 1 |
|  | *Mycoplasma* | 15 | 0 | 1 | 1 |
|  | *Neoehrlichia mikurensis* | 15 | 0 | 1 | 1 |
|  | *Rickettsia aeschlimannii* | 15 | 0 | 1 | 1 |
|  | *Rickettsia conorii* | 15 | 0 | 1 | 1 |
|  | *Rickettsia slovaca* | 15 | 0 | 1 | 1 |
|  | *Rickettsia* | 15 | 0 | 1 | 1 |
|  | *Theleiria* | 15 | 0 | 1 | 1 |
| FW | *Anaplasma phagocytophilum* | 11 | 0 | 1 | 1 |
|  | *Anaplasma* | 11 | 0 | 1 | 1 |
|  | Apicomplexa | 11 | 0 | 1 | 1 |
|  | *Borrelia afzelii* | 11 | 0 | 1 | 1 |
|  | *Borrelia spielmanii* | 11 | 0 | 1 | 1 |
|  | *Francisella like endosymbiont* | 11 | 0 | 1 | 1 |
|  | *Neoehrlichia mikurensis* | 11 | 0 | 1 | 1 |
|  | *Rickettsia aeschlimannii* | 11 | 0 | 1 | 1 |
|  | *Rickettsia conorii* | 11 | 0 | 1 | 1 |
|  | *Rickettsia slovaca* | 11 | 0 | 1 | 1 |
|  | *Rickettsia* | 11 | 0 | 1 | 1 |
| FSP | Apicomplexa | 6 | 0 | 1 | 1 |
|  | *Borrelia spielmanii* | 6 | 0 | 1 | 1 |
|  | *Francisella like endosymbiont* | 6 | 0 | 1 | 1 |
|  | *Neoehrlichia mikurensis* | 6 | 0 | 1 | 1 |
|  | *Rickettsia conorii* | 6 | 0 | 1 | 1 |
|  | *Rickettsia slovaca* | 6 | 0 | 1 | 1 |
|  | *Rickettsia* | 6 | 0 | 1 | 1 |
| FSU | *Anaplasma* | 13 | 0 | 1 | 1 |
|  | Apicomplexa | 13 | 0 | 1 | 1 |
|  | *Bartonella* | 13 | 0 | 1 | 1 |
|  | *Borrelia afzelii* | 13 | 0 | 1 | 1 |
|  | *Borrelia spielmanii* | 13 | 0 | 1 | 1 |
|  | *Francisella like endosymbiont* | 13 | 0 | 1 | 1 |
|  | *Francisella tularensis* | 13 | 0 | 1 | 1 |
|  | *Hepatozoon* | 13 | 0 | 1 | 1 |
|  | *Mycoplasma* | 13 | 0 | 1 | 1 |
|  | *Neoehrlichia mikurensis* | 13 | 0 | 1 | 1 |
|  | *Rickettsia conorii* | 13 | 0 | 1 | 1 |
|  | *Rickettsia slovaca* | 13 | 0 | 1 | 1 |
|  | *Rickettsia* | 13 | 0 | 1 | 1 |
|  | *Theleiria* | 13 | 0 | 1 | 1 |
| FA | *Anaplasma* | 12 | 0 | 1 | 1 |
|  | Apicomplexa | 12 | 0 | 1 | 1 |
|  | *Borrelia afzelii* | 12 | 0 | 1 | 1 |
|  | *Borrelia spielmanii* | 12 | 0 | 1 | 1 |
|  | *Francisella like endosymbiont* | 12 | 0 | 1 | 1 |
|  | *Hepatozoon* | 12 | 0 | 1 | 1 |
|  | *Mycoplasma* | 12 | 0 | 1 | 1 |
|  | *Neoehrlichia mikurensis* | 12 | 0 | 1 | 1 |
|  | *Rickettsia aeschlimannii* | 12 | 0 | 1 | 1 |
|  | *Rickettsia conorii* | 12 | 0 | 1 | 1 |
|  | *Rickettsia slovaca* | 12 | 0 | 1 | 1 |
|  | *Rickettsia* | 12 | 0 | 1 | 1 |
|  | *Theleiria* | 12 | 0 | 1 | 1 |
