# Supplementary material for: Guild Dynamics and Pathogen Interactions in Hyalomma Ticks From Algerian Cattle
Source: Transbound Emerg Dis. 2024 Dec 7;2024:5384559. doi: 10.1155/tbed/5384559 (PMC12016691; doi:10.1155/tbed/5384559)
Supplement: Supporting Information 6 — Table S5: Centrality measures of shared pathogens across TBPGs networks. [file 5384559.f6.docx]

**Supplementary Table S5.** Centrality measures of shared pathogens across Tick-Borne Pathogen Guilds (TBPGs).

| Pathogen (TBP) | TBPG Identifier | Degree Centrality | Betweenness Centrality | Eigenvector Centrality | Closeness Centrality | Notes |
| --- | --- | --- | --- | --- | --- | --- |
| *Anaplasma phagocytophilum* | F | 15 | 0 | 1 | 1 |  |
|  | FW | 11 | 0 | 1 | 1 |  |
| *Anaplasma* | M | 11 | 0 | 1 | 1 |  |
|  | MA | 8 | 0 | 1 | 1 |  |
|  | F | 15 | 0 | 1 | 1 |  |
|  | FW | 11 | 0 | 1 | 1 |  |
|  | FSU | 13 | 0 | 1 | 1 |  |
|  | FA | 12 | 0 | 1 | 1 |  |
| Apicomplexa | M | 11 | 0 | 1 | 1 |  |
|  | MW | 3 | 0 | 1 | 1 |  |
|  | MSP | 5 | 0 | 1 | 1 |  |
|  | MSU | 6 | 0 | 1 | 1 |  |
|  | F | 15 | 0 | 1 | 1 |  |
|  | FW | 11 | 0 | 1 | 1 |  |
|  | FSP | 6 | 0 | 1 | 1 |  |
|  | FSU | 13 | 0 | 1 | 1 |  |
| *Coxiella like endosymbiont* | M | 11 | 0 | 1 | 1 |  |
|  | MSP | 5 | 0 | 1 | 1 |  |
| *Bartonella* | M | 11 | 0 | 1 | 1 |  |
|  | MSP | 5 | 0 | 1 | 1 |  |
|  | F | 15 | 0 | 1 | 1 |  |
|  | FSU | 13 | 0 | 1 | 1 |  |
| *Borrelia afzelii* | F | 15 | 0 | 1 | 1 |  |
|  | FW | 11 | 0 | 1 | 1 |  |
|  | FSU | 13 | 0 | 1 | 1 |  |
|  | FA | 12 | 0 | 1 | 1 |  |
| *Borrelia spielmanii* | F | 15 | 0 | 1 | 1 |  |
|  | FW | 11 | 0 | 1 | 1 |  |
|  | FSP | 6 | 0 | 1 | 1 |  |
|  | FSU | 13 | 0 | 1 | 1 |  |
|  | FA | 12 | 0 | 1 | 1 |  |
| *Ehrilichia* | M | 11 | 0 | 1 | 1 |  |
|  | MA | 8 | 0 | 1 | 1 |  |
| *Francisella like endosymbiont* | M | 11 | 0 | 1 | 1 |  |
|  | MW | 3 | 0 | 1 | 1 |  |
|  | MSP | 5 | 0 | 1 | 1 |  |
|  | MSU | 6 | 0 | 1 | 1 |  |
|  | MA | 8 | 0 | 1 | 1 |  |
|  | F | 15 | 0 | 1 | 1 |  |
|  | FW | 11 | 0 | 1 | 1 |  |
|  | FSP | 6 | 0 | 1 | 1 |  |
|  | FSU | 13 | 0 | 1 | 1 |  |
|  | FA | 12 | 0 | 1 | 1 |  |
| *Francisella tularensis* | M | 11 | 0 | 1 | 1 |  |
|  | MA | 8 | 0 | 1 | 1 |  |
|  | F | 15 | 0 | 1 | 1 |  |
|  | FSU | 13 | 0 | 1 | 1 |  |
| *Hepatozoon* | F | 15 | 0 | 1 | 1 |  |
|  | FSU | 13 | 0 | 1 | 1 |  |
|  | FA | 12 | 0 | 1 | 1 |  |
| *Mycoplasma* | F | 15 | 0 | 1 | 1 |  |
|  | FSU | 13 | 0 | 1 | 1 |  |
|  | FA | 12 | 0 | 1 | 1 |  |
| *Neoehrlichia mikurensis* | M | 11 | 0 | 1 | 1 |  |
|  | MA | 8 | 0 | 1 | 1 |  |
|  | F | 15 | 0 | 1 | 1 |  |
|  | FW | 11 | 0 | 1 | 1 |  |
|  | FSP | 6 | 0 | 1 | 1 |  |
|  | FSU | 13 | 0 | 1 | 1 |  |
|  | FA | 12 | 0 | 1 | 1 |  |
| *Rickettsia aeschlimannii* | M | 11 | 0 | 1 | 1 |  |
|  | MA | 8 | 0 | 1 | 1 |  |
|  | F | 15 | 0 | 1 | 1 |  |
|  | FW | 11 | 0 | 1 | 1 |  |
|  | FA | 12 | 0 | 1 | 1 |  |
| *Rickettsia conorii* | M | 11 | 0 | 1 | 1 |  |
|  | MSP | 5 | 0 | 1 | 1 |  |
|  | MSU | 6 | 0 | 1 | 1 |  |
|  | MA | 8 | 0 | 1 | 1 |  |
|  | F | 15 | 0 | 1 | 1 |  |
|  | FW | 11 | 0 | 1 | 1 |  |
|  | FSP | 6 | 0 | 1 | 1 |  |
|  | FSU | 13 | 0 | 1 | 1 |  |
|  | FA | 12 | 0 | 1 | 1 |  |
| *Rickettsia slovaca* | M | 11 | 0 | 1 | 1 |  |
|  | MW | 3 | 0 | 1 | 1 |  |
|  | MSP | 5 | 0 | 1 | 1 |  |
|  | MSU | 6 | 0 | 1 | 1 |  |
|  | MA | 8 | 0 | 1 | 1 |  |
|  | F | 15 | 0 | 1 | 1 |  |
|  | FW | 11 | 0 | 1 | 1 |  |
|  | FSP | 6 | 0 | 1 | 1 |  |
|  | FSU | 13 | 0 | 1 | 1 |  |
|  | FA | 12 | 0 | 1 | 1 |  |
| *Rickettsia* | M | 11 | 0 | 1 | 1 |  |
|  | MW | 3 | 0 | 1 | 1 |  |
|  | MSP | 5 | 0 | 1 | 1 |  |
|  | MSU | 6 | 0 | 1 | 1 |  |
|  | MA | 8 | 0 | 1 | 1 |  |
|  | F | 15 | 0 | 1 | 1 |  |
|  | FW | 11 | 0 | 1 | 1 |  |
|  | FSP | 6 | 0 | 1 | 1 |  |
|  | FSU | 13 | 0 | 1 | 1 |  |
|  | FA | 12 | 0 | 1 | 1 |  |
| *Theleiria* | M | 11 | 0 | 1 | 1 |  |
|  | MA | 8 | 0 | 1 | 1 |  |
|  | F | 15 | 0 | 1 | 1 |  |
|  | FA | 12 | 0 | 1 | 1 |  |
